# Discovery and Genomic Characterization of a 382-Nucleotide Deletion in ORF7b and ORF8 during the Early Evolution of SARS-CoV-2

**DOI:** 10.1128/mBio.01610-20

**Published:** 2020-07-21

**Authors:** Yvonne C. F. Su, Danielle E. Anderson, Barnaby E. Young, Martin Linster, Feng Zhu, Jayanthi Jayakumar, Yan Zhuang, Shirin Kalimuddin, Jenny G. H. Low, Chee Wah Tan, Wan Ni Chia, Tze Minn Mak, Sophie Octavia, Jean-Marc Chavatte, Raphael T. C. Lee, Surinder Pada, Seow Yen Tan, Louisa Sun, Gabriel Z. Yan, Sebastian Maurer-Stroh, Ian H. Mendenhall, Yee-Sin Leo, David Chien Lye, Lin-Fa Wang, Gavin J. D. Smith

**Affiliations:** a Programme in Emerging Infectious Diseases, Duke-NUS Medical School, Singapore; b National Centre for Infectious Diseases, Singapore; c Tan Tock Seng Hospital, Singapore; d Lee Kong Chian School of Medicine, Singapore; e Singapore General Hospital, Singapore; f Bioinformatics Institute, Agency for Science, Technology and Research (A*STAR), Singapore; g Ng Teng Fong General Hospital, Singapore; h Changi General Hospital, Singapore; i Alexandra Hospital, Singapore; j National University Hospital, Singapore; k Department of Biological Sciences, National University of Singapore, Singapore; l Saw Swee Hock School of Public Health, National University of Singapore, Singapore; m SingHealth Duke-NUS Global Health Institute, Singapore; n Yong Loo Lin School of Medicine, National University of Singapore, Singapore; o Duke Global Health Institute, Duke University, North Carolina, USA; St. Jude Children's Research Hospital

**Keywords:** COVID-19, ORF8, natural selection, phylogeny, vaccines

## Abstract

To date, limited genetic changes in the severe acute respiratory syndrome coronavirus 2 (SARS-CoV-2) genome have been described. Here, we report a 382-nucleotide (nt) deletion in SARS-CoV-2 that truncates open reading frame 7b (ORF7b) and ORF8, removing the ORF8 transcription regulatory sequence (TRS) and eliminating ORF8 transcription. The earliest 382-nt deletion variant was detected in Singapore on 29 January 2020, with the deletion viruses circulating in the country and accounting for 23.6% (45/191) of SARS-CoV-2 samples screened in this study. SARS-CoV-2 with the same deletion has since been detected in Taiwan, and other ORF7b/8 deletions of various lengths, ranging from 62 nt to 345 nt, have been observed in other geographic locations, including Australia, Bangladesh, and Spain. Mutations or deletions in ORF8 of SARS-CoV have been associated with reduced replicative fitness and virus attenuation. In contrast, the SARS-CoV-2 382-nt deletion viruses showed significantly higher replicative fitness *in vitro* than the wild type, while no difference was observed in patient viral load, indicating that the deletion variant viruses retained their replicative fitness. A robust antibody response to ORF8 has been observed in SARS-CoV-2 infection, suggesting that the emergence of ORF8 deletions may be due to immune-driven selection and that further deletion variants may emerge during the sustained transmission of SARS-CoV-2 in humans.

## Abstract (precis)

During the SARS epidemic in 2003/2004, a number of deletions were observed in ORF8 of SARS-CoV, and eventually deletion variants became predominant, leading to the hypothesis that ORF8 was an evolutionary hot spot for adaptation of SARS-CoV to humans. However, due to the successful control of the SARS epidemic, the importance of these deletions for the epidemiological fitness of SARS-CoV in humans could not be established. The emergence of multiple SARS-CoV-2 strains with ORF8 deletions, combined with evidence of a robust immune response to ORF8, suggests that the lack of ORF8 may assist with host immune evasion. In addition to providing a key insight into the evolutionary behavior of SARS-CoV-2 as the virus adapts to its new human hosts, the emergence of ORF8 deletion variants may also impact vaccination strategies.

## INTRODUCTION

In December 2019, a novel coronavirus (CoV) emerged from Hubei province in China and infected people visiting the Huanan seafood market in Wuhan (1). The virus demonstrated efficient human-to-human transmission within mainland China and subsequently spread across many countries. The virus was soon identified as novel CoV 2019 (2019-nCoV), more recently designated severe acute respiratory syndrome coronavirus 2 (SARS-CoV-2), while the associated disease is referred to as coronavirus disease 2019 (COVID-19). On 30 January 2020, the World Health Organization declared a Public Health Emergency of International Concern. As of 15 June 2020, there were almost 8 million confirmed COVID-19 cases and 431,541 deaths globally (2). In Singapore, the first case of SARS-CoV-2 was reported on 23 January 2020, in a 66-year-old man that had visited Wuhan. By 7 February, the number of cases had increased to 29, including individuals without travel history to China, prompting Singaporean authorities to implement control measures aimed at reducing the community spread of the virus (3). Successful zoonotic viral transmission from animals to humans is often associated with the ability of viruses to adapt to a new host, via genetic mutation events, and cause sustained transmission (4–6). A variety of genomic changes, including mutations, deletions, and recombinations, have been frequently observed in the other two documented zoonotic coronaviruses, SARS-CoV and Middle East respiratory syndrome-coronavirus (MERS-CoV) (7, 8).

## RESULTS

**FIG 1:**
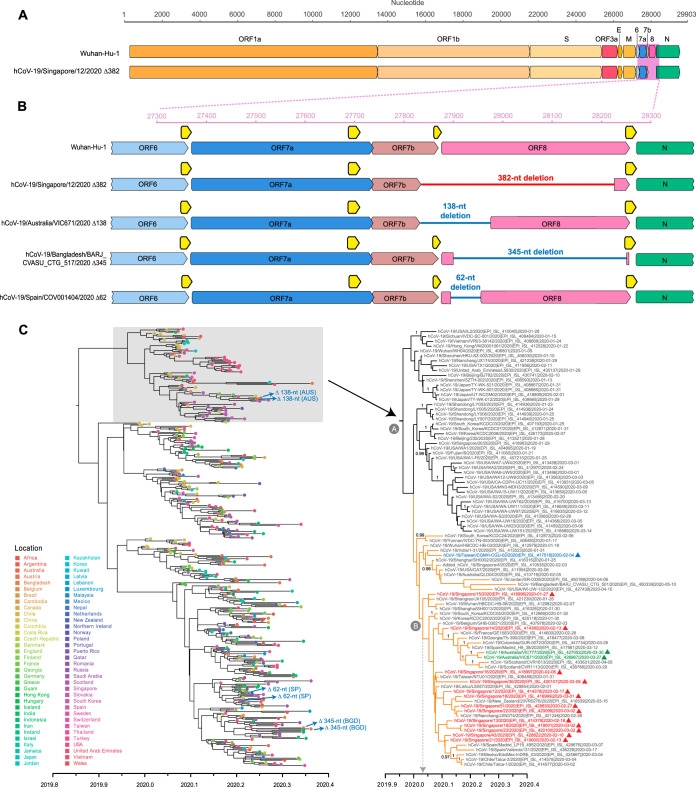
Genomic organization and evolutionary relationships of human SARS-CoV-2 and SARS-CoV-2 Δ382. (A) Schematic diagram of the genomes of SARS-CoV-2 isolates Wuhan-Hu-1 (GenBank accession no. MN908947) and human CoV-19/Singapore/12/2020 (hCoV-19/Singapore/12/2020) Δ382 (GISAID: EPI_ISL_414378). (B) Magnification of genomic region (pink box in panel A) showing the 382-nt deletion in ORF7b and ORF8 (indicated by red line) in hCoV-19/Singapore/12/2020. Other ORF7b/8 deletions are indicated by blue lines as follows: a 138-nt deletion in hCoV-19/Australia/VIC671/2020 (EPI_ISL_426967), a 345-nt deletion in hCoV-19/Bangladesh/BARJ_CVASU_CTG_517/2020 (EPI_ISL_450344), and a 62-nt deletion in hCoV-19/Spain/COV001404/2020 (EPI_ISL_452497). Horizontal axes indicate the nucleotide position relative to Wuhan-Hu-1; open reading frames (ORFs) are indicated by solid colored arrows. Transcription regulatory sequences (TRSs) are indicated by yellow arrows. (C) Temporal phylogeny of 419 complete genomes inferred using an uncorrelated lognormal relaxed clock model in BEAST. Colored circles at the tips represent geographic locations of virus sampling. Colored triangles represent ORF7b/8 deletion variants. A fully labeled tree with Bayesian posterior probabilities indicated is presented in Fig. S4. Red isolate names indicate SARS-CoV-2 from Singapore with a 382-nt deletion as described in this study. Blue isolate names indicate a 382-nt deletion from Taiwan, whereas green isolate names indicate a 138-nt deletion from Australia. Node A represents the time to most common ancestor (TMRCA) for the lineage containing Δ382 viruses from Singapore and Taiwan, while node B represents the TMRCA of the clade containing Δ382 viruses from Singapore. Bayesian posterior probabilities of ≥0.95 are indicated at nodes. Scale bar represents time in years. Abbreviations: AUS, Australia; SP, Spain; BGD, Bangladesh.

During the early epidemic in January to March 2020, we collected clinical specimens (including nasopharyngeal swab, endotracheal aspirate, urine, and stool samples) from 28 hospitalized patients that tested positive for SARS-CoV-2 in Singapore. Specimens were subjected to metagenomic next-generation sequencing (NGS), with and without passaging in Vero-E6 cells, and 21 full genomes were recovered (see Table S1 in the supplemental material). Whole-genome sequencing of early samples showed that SARS-CoV-2 from Singapore shared high similarity with viruses from Wuhan, which was expected due to the importation of cases from China. Sequencing of later patient samples showed a large deletion toward the 3′ end in 6 of the 21 virus genomes (Fig. 1A). To verify this observation, specific PCR primers flanking the deleted region were designed and Sanger sequencing confirmed a 382-nucleotide (nt) deletion corresponding to positions 27,848 to 28,229 of the SARS-CoV-2 genome (see Fig. S1 in the supplemental material). Interrogation of the NGS assemblies of these 382-nt deletion variants (referred to here as Δ382) indicated that the virus populations were homogenous. Apart from the 382-nt deletion, the genome organization of the Δ382 viruses is identical to that of other SARS-CoV-2 isolates (Fig. 1A). Closer examination indicated that the deletion spans an area of open reading frame 7b (ORF7b) and ORF8 (Fig. 1B). Specifically, there is a 40-nt deletion at the 3′ end of ORF7b, a deletion of the 6-nt intergenic region of ORF7b/8, and a 336-nt deletion from the 5′ end of ORF8 that eliminates the ORF8 transcription regulatory sequence (TRS). The 382-nt deletion also results in a predicted ORF7b-ORF8 fusion protein composed of a truncated ORF7b in which its C-terminal 12 amino acids (aa), including the stop codon, are replaced with the last 5 aa from the remaining C terminus of ORF8 (Fig. S2).

10.1128/mBio.01610-20.1 FIG S1 Sanger sequences of hCoV-19/Singapore/12/2020 Δ382 mapped to Wuhan-Hu-1 showing the position of the 382-nt deletion in the ORF7b and ORF8 regions of the SARS-CoV-2 genome. Download FIG S1, PDF file, 0.7 MB. Copyright © 2020 Su et al. 2020 Su et al. This content is distributed under the terms of the Creative Commons Attribution 4.0 International license.

10.1128/mBio.01610-20.2 FIG S2 Amino acid sequences of the ORF7b region in SARS-CoV-2 and SARS-CoV-2 Δ382. Truncated ORF7b is observed as a result of the deleted C terminus of ORF7b in SARS-CoV-2 Δ382. Download FIG S2, PDF file, 0.4 MB. Copyright © 2020 Su et al. 2020 Su et al. This content is distributed under the terms of the Creative Commons Attribution 4.0 International license.

10.1128/mBio.01610-20.6 TABLE S1 List of specimens from which full genomes were generated. Download Table S1, DOCX file, 0.01 MB. Copyright © 2020 Su et al. 2020 Su et al. This content is distributed under the terms of the Creative Commons Attribution 4.0 International license.

To investigate the possible origin and evolutionary relationships of these Δ382 viruses, a maximum likelihood (ML) phylogeny of SARS-CoV-2 full genomes was inferred from serially sampled data sets. The global evolutionary tree shows the cocirculation of multiple lineages (Fig. S3), consistent with published topologies (9), indicating lineage diversification of this pandemic virus following zoonotic transmission. The ML tree indicates that all Δ382 viruses from Singapore (*n* = 11), plus an additional Δ382 virus genome from Taiwan (*n* = 1), formed a monophyletic clade (Fig. S3), although there is a lack of statistical support, reflecting that these Δ382 viruses share a high (99.9%) level of nucleotide similarity (Table S2). We then inferred time-scaled phylogenies of SARS-CoV-2 using a relaxed-molecular-clock model in BEAST. The dated tree demonstrates the intra- and intercontinental dissemination of the wild-type (WT) viruses, whereas all Δ382 viruses from Singapore (marked by red triangles in Fig. 1C) and Taiwan (marked by a green triangle) are closely related; however, there is a lack of statistical support. While it is not possible to determine the direction of transmission based on the phylogeny, our results suggest that the introduction of Δ382 viruses likely arose from a single source rather than from multiple introductions of variants into Singapore.

10.1128/mBio.01610-20.3 FIG S3 Maximum-likelihood tree of SARS-CoV-2 genomes (*n* = 1,038) reconstructed using RAxML with bootstrap values of >50 indicated at branch nodes. Download FIG S3, PDF file, 0.3 MB. Copyright © 2020 Su et al. 2020 Su et al. This content is distributed under the terms of the Creative Commons Attribution 4.0 International license.

10.1128/mBio.01610-20.4 FIG S4 Evolutionary relationships of human SARS-CoV-2 and SARS-CoV-2 Δ382. Temporal phylogeny of 419 complete genomes was inferred using an uncorrelated lognormal relaxed clock model in BEAST. Colored virus names represent different geographic locations. Bayesian posterior probabilities of ≥0.95 are indicated at nodes. The scale bar represents time in years. Download FIG S4, PDF file, 0.3 MB. Copyright © 2020 Su et al. 2020 Su et al. This content is distributed under the terms of the Creative Commons Attribution 4.0 International license.

10.1128/mBio.01610-20.7 TABLE S2 Comparison of genome similarities between SARS-CoV-2 Δ382 viruses and the wild-type virus Wuhan-Hu-1 SARS-CoV-2. Download Table S2, DOCX file, 0.01 MB. Copyright © 2020 Su et al. 2020 Su et al. This content is distributed under the terms of the Creative Commons Attribution 4.0 International license.

Our date estimates indicate the Δ382 viruses emerged between the middle of December 2019 (node A time to most common ancestor [TMRCA] 95% highest posterior density [HPD], 2019.90 to 2020.00) and early January 2020 (node B TMRCA 95% HPD, 2019.98 to 2020.04) (Fig. 1C; see also Table S3), suggesting rapid mutation of SARS-CoV-2 following its emergence. Consistent with previous analyses, the estimated rate of nucleotide substitutions among SARS-CoV-2 viruses is approximately 0.91 × 10^−3^ substitutions per site per year (95% HPD, 0.79 × 10^−3^ to 1.03 × 10^−3^) and dating estimates indicate that the introduction of SARS-CoV-2 into humans occurred early November 2019 (TMRCA 95% HPD, 2019.76 to 2019.92) (Table S3), suggesting that the viruses were present in humans approximately 1 month before the outbreak was detected.

10.1128/mBio.01610-20.8 TABLE S3 Estimated dates of nodes within the SARS-CoV-2 phylogeny shown in Fig. 1C. Download Table S3, DOCX file, 0.01 MB. Copyright © 2020 Su et al. 2020 Su et al. This content is distributed under the terms of the Creative Commons Attribution 4.0 International license.

In addition to Δ382 viruses, other ORF7b and/or ORF8 deletions, from 62 nt to 345 nt, have been observed in Australia (*n* = 2), Bangladesh (*n* = 2), and Spain (*n* = 2) (Fig. 1B; see also Table S4). Singapore and Taiwan Δ382 viruses are grouped with Australia Δ138 viruses within the same lineage (lineage A), whereas Bangladesh Δ345 viruses and Spain Δ62 viruses fall in lineage B (Fig. S3). The Δ138 viruses from Australia form a strongly supported monophyletic group (Bayesian posterior probability = 1.00) and appear to be closely related to viruses circulating from Europe (Fig. 1C; see also Fig. S3). The 138-nt deletion occurs from nucleotide position 27,846 to position 27983 of the SARS-CoV-2 genomes, resembling Singapore Δ382 viruses with nucleotide deletions across ORF7b and ORF8 regions (Fig. 1B), although the remaining ORF8 of Δ138 variants remains intact.

10.1128/mBio.01610-20.9 TABLE S4 Summary of deletion variants of SARS-CoV-2. Download Table S4, DOCX file, 0.01 MB. Copyright © 2020 Su et al. 2020 Su et al. This content is distributed under the terms of the Creative Commons Attribution 4.0 International license.

**FIG 2:**
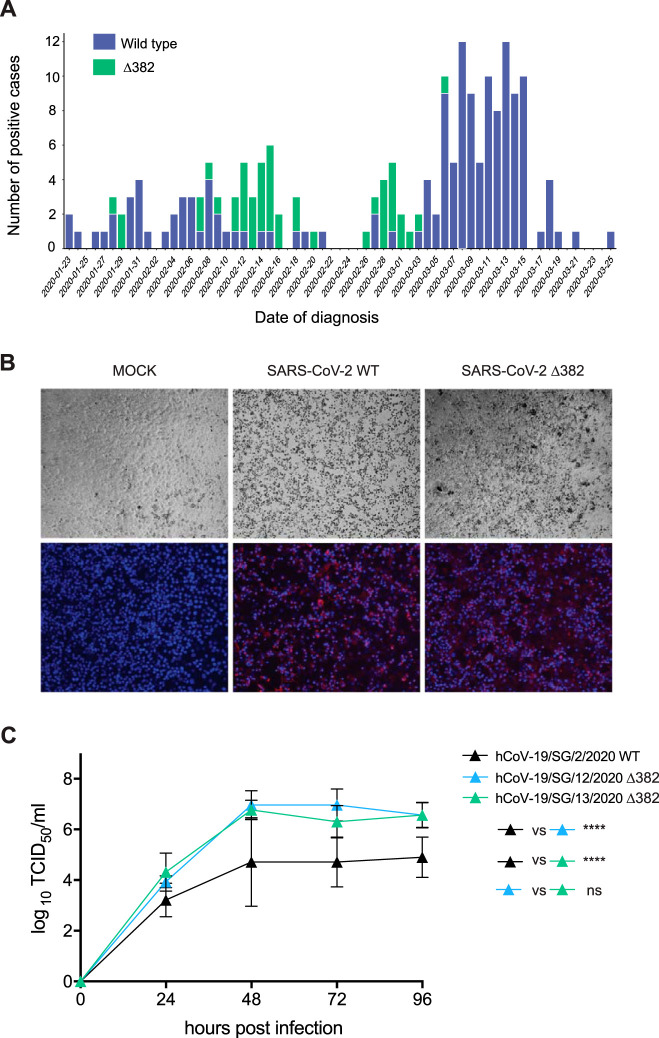
Prevalence, viral antigen staining, and growth kinetics of human SARS-CoV-2 and SARS-CoV-2 Δ382. (A) Daily number of wild-type and Δ382 deletion variants of SARS-CoV-2 detected in Singapore based on PCR screening of 191 patient specimens. (B) Cellular and viral fluorescence staining of SARS-CoV-2 wild-type and Δ382 strains in Vero-E6 cells. Bright-field images were captured to visualize cytopathic effect. SARS-CoV-2 antigen was stained with COVID-19 convalescent-phase serum. Red, viral proteins; blue, DAPI (nuclei). (C) Growth curves of SARS-CoV-2 wild-type and Δ382 strains at a multiplicity of infection (MOI) of 0.01 in Vero-E6 cells. Virus titers were expressed as 50% tissue culture infectious doses (TCID_50_)/ml and were plotted as means of results from three independent replicates and standard deviations. Error bars indicate standard errors of means. Significance was calculated by two-way ANOVA with Tukey’s multiple-comparison test.

We then tested residual diagnostic samples from COVID-19 patients in Singapore for the presence of Δ382 viruses to understand the epidemiological fitness of Δ382 viruses. Using in-house PCR primers and Sanger sequencing, we screened a total of 191 individual samples collected from 23 January 2020 to 25 March 2020, among which 45 (23.6%) contained the 382-nt deletion (Fig. 2A), suggesting that these viruses retain the ability to efficiently infect and transmit between humans. This is further supported by evaluation of the growth of Δ382 SARS-CoV-2 viruses *in vitro* and by patient viral load data. We compared two Singapore Δ382 isolates with the wild type using Vero-E6 cells. While Δ382 SARS-CoV-2 displayed replication kinetics similar to the wild-type kinetics at 24 h postinfection (hpi), titers of the Δ382 viruses were significantly higher at later time points, even though the cytopathic effects were similar (Fig. 2B and C). The viral loads seen in nasopharyngeal samples from patients infected with SARS-CoV-2 WT virus (*n* = 8, 22.48 ± 3.97) were not significantly different (*P* = 0.175) from those obtained from Δ382 virus-infected individuals (*n* = 11, 27.00 ± 6.31). This suggests that despite the loss of partial ORF7b and ORF8 regions in the genome, SARS-CoV-2 Δ382 viruses retain replicative fitness *in vitro* and *in vivo*. The earliest Δ382 viruses were detected on 28 and 29 January 2020 from individuals that had recently traveled from Wuhan, China, coinciding with our estimated date of introduction and indicating the likely origin of the Δ382 virus. The most recent Δ382 viruses were detected in Singapore on 6 March 2020, suggesting that these viruses are no longer circulating in Singapore, likely due to the aggressive contact tracing and isolation/quarantine that had been enacted in the country. Our data, together with the detection of viruses with an identical 382-nt deletion in Taiwan, indicates that Δ382 viruses are fit and capable of transmission within humans, raising the possibility that more Δ382 viruses may be discovered when additional genomic data become available.

## DISCUSSION

A number of genomic deletions in SARS-CoV were observed during the course of the 2003/2004 SARS epidemic (7, 10–15). A 29-nt ORF8 deletion occurred in all SARS-CoVs in the middle and late phases, while complete or nearly complete ORF8 deletions were observed toward the end of the outbreak (7, 13, 15). The gradual occurrence of these deletions and their eventual predominance led to the hypothesis that ORF8 was an evolutionary hot spot for adaptation of SARS-CoV to humans (7, 11, 13). Experimental studies have since shown that ORF8 of SARS-CoV plays a functional role in virus replicative fitness *in vitro*, with partial or full deletions of ORF8 demonstrating reduced replication compared to the wild type (16). However, full-length ORF8 of human SARS-CoV is only distantly related to that of SAR-CoV-2 (55.4% nt similarity), and the genomic features are correspondingly divergent. For example, ORF8 of SARS-CoV-2 lacks a functional motif (VLVVL) present in SARS-CoV ORF8b (17) that is associated with induction of cell stress pathways and activation of macrophages during SARS-CoV infection (18). Further investigation is required to determine whether the predicted ORF7b-ORF8 fusion protein (see Fig. S2 in the supplemental material) is translated and whether it has any associated virus phenotype, although human SARS-CoV (Frankfurt-1 strain) with an ORF7b deletion shows higher growth *in vitro* than viruses with the full-length ORF7b (19). A comparison of subgenomic RNA reads predicted from the sequence data (see the supplemental material) suggests that Δ382 viruses may have altered levels of transcription compared to wild-type viruses (Fig. S5), including those of the ORF6 and N genes which are known SARS-CoV interferon (IFN) antagonists (20–23), raising the possibility that infection with Δ382 viruses might result in an altered innate immune response. Due to the successful control of the SARS epidemic, the importance of these deletions for the epidemiological fitness of SARS-CoV in humans remains unknown, and experimental studies are required to assess any virus phenotypic changes in SARS-CoV-2 due to the 382-nt and other ORF8 deletions.

10.1128/mBio.01610-20.5 FIG S5 Comparison of data representing transcription of different SARS-CoV-2 genes in wild-type (WT) versus Δ382 viruses. The abundance of mapped reads relative to transcription regulatory sequence (TRS) positions across the genome was determined. Transcripts per million (TPM) reads were calculated from reads mapped specifically to each leader-TRS region, and a whisker and a scatter plot was drawn for each gene. A Wilcoxon test was applied to the TPM data for comparison of each gene of Δ382 to the WT (*, *P* ≤ 0.05; **, *P* ≤ 0.01). Download FIG S5, PDF file, 0.2 MB. Copyright © 2020 Su et al. 2020 Su et al. This content is distributed under the terms of the Creative Commons Attribution 4.0 International license.

In this report, we describe a major evolutionary event of the SARS-CoV-2 virus following its emergence in humans. Although the biological consequences of this deletion remain largely unknown, the observed replication differences and previously described immunological consequences of SARS-CoV genome deletions suggest potential phenotypic changes in Δ382 viruses. The robust immune response to ORF8 during SARS-CoV-2 infection (24) also suggests that the emergence of ORF8 deletions may be due to immune-driven selection. Given that genetic variants will continue to arise driven by random mutation and natural selection, it is likely that we will see further deletion variants emerge with the sustained transmission of SARS-CoV-2 in humans. Although metagenomics can provide advanced tools to track the changing dynamics of SARS-CoV-2, the complex mechanisms underpinning pathogenicity, epidemiological behavior, transmission patterns, and host immunity must be examined to provide a more comprehensive understanding of this unfolding disease outbreak.

## MATERIALS AND METHODS

### Ethics statement.

This study was undertaken as part of the national disease outbreak, and the response and the protocols were approved by the ethics committee of the National Healthcare Group. Patient samples were collected under the guidelines provided by PROTECT (2012/00917), a multicentered prospective study to detect novel pathogens and characterize emerging infections. Work undertaken at the Duke-NUS Medical School animal biological safety level 3 (ABSL-3) laboratory was approved by the Duke-NUS ABSL3 Biosafety Committee, the National University of Singapore, and the Ministry of Health Singapore.

### Virus culture, RNA extraction, and sequencing.

Clinical samples from SARS-CoV-2-positive patients were collected at public hospitals in Singapore from January through February 2020. Clinical samples were used to inoculate Vero-E6 cells (ATCC CRL-1586). Total RNA was extracted using E.Z.N.A. total RNA kit I (Omega Bio-Tek) according to the manufacturer’s instructions, and the samples were analyzed by real-time quantitative reverse transcription-PCR for the detection of SARS-CoV-2 as previously described (25). Whole-genome sequencing was performed using next-generation sequencing (NGS) methodology. The cDNA libraries were constructed using a TruSeq RNA library prep kit (Illumina) according to the manufacturer’s instructions and sequenced on an Illumina MiSeq system. Raw NGS reads were trimmed by the use of Trimmomatic v0.39 (26) to remove adaptors and low-quality bases. Genome sequences were assembled and consensus sequences obtained using the BWA-MEM algorithm in UGENE v.33. To verify the presence of the deletion in the SARS-CoV-2 genome, we designed two specific PCR primers (primer F [5′-TGTTAGAGGTACAACAGTACTTT-3′] and primer R [5′-GGTAGTAGAAATACCATCTTGGA-3′]) targeting the ORF7-to-ORF8 regions. For samples with low cycle threshold (*C_T_*) values, a second heminested PCR was performed with primers 5′-TGTTTATAACACTTTGCTTCACA-3′ and 5′-GGTAGTAGAAATACCATCTTGGA-3′. The PCR mixture contained the cDNA, primers (10 μM each), 10× *Pfu* reaction buffer (Promega), *Pfu* DNA polymerase (Promega), and deoxynucleoside triphosphate (dNTP) mix (Thermo Scientific) (10 mM). The PCR was carried out under the following conditions: 95°C for 2 min; 35 cycles at 95°C for 1 min, 52°C for 30 s, and 72°C for 1 min; and a final extension at 72°C for 10 min in a thermal cycler (Applied Biosystems Veriti). Deletions in the PCR products were visualized by gel electrophoresis and confirmed by Sanger sequencing. Full complete genomes of SARS-CoV-2 wild-type and Δ382 viruses generated in Singapore were deposited in the GISAID database (see Table S1 in the supplemental material).

### Genomic characterization.

To characterize and map the deletion regions of SARS-CoV-2 viruses, we compared viral genome organizations of Wuhan-Hu-1 (GenBank accession number MN908947) and Singapore SARS-CoV-2 (Singapore/2/2020: EPI_ISL_407987). The genomes comprised the following gene order and lengths: ORF1ab (open-reading frame) replicase (21,291 nt), spike (S: 3,822 nt), ORF3 (828 nt), envelope (E: 228 nt), membrane (M: 669 nt), ORF6 (186 nt), ORF7ab (498 nt), ORF8 (366 nt), nucleocapsid (N: 1,260 nt), and ORF10 (117 nt).

### Phylogenetic analyses.

All available genomes of SARS-CoV-2 with associated virus sampling dates were downloaded from the GISAID database. To reduce bias from locations with higher virus sampling and genome availability, data sets were subsampled randomly based on geographical location and collection month using in-house scripts. Genome sequence alignment was performed using MAFFT (27) in Geneious R9.0.3 software (Biomatters Ltd.) followed by manual alignment. Maximum likelihood phylogenies of 1,038 complete genomes were reconstructed using RAxML with 200 bootstrap replicates (28). Any sequence outliers were removed from subsequent analyses. Lineage circumscription of SARS-CoV-2 was conducted using pangolin software (9). To reconstruct a time-scaled phylogeny, we analyzed serially sampled data sets of 419 complete genomes (Table S5) using an uncorrelated lognormal relaxed-clock (UCLN) model with an exponential growth coalescent prior and the HKY85+Γ substitution model in BEAST program v1.10.4 (29) to simultaneously estimate phylogenies, divergence times, and rates of nucleotide substitution. The strict and UCLN models were recently tested by Duchene et al. (30), who showed that the UCLN model is preferred over a strict clock for analyzing large (>122) SARS-CoV-2 genome data sets. At least four independent Markov chain Monte Carlo (MCMC) runs of 100 million generations were performed with sampling every 10,000 generations. The runs were checked for convergence in Tracer v1.7 (31), and the effective sampling size (ESS) values of all parameters were >200. The resulting log and tree files were combined after removal of appropriate burn-in values using LogCombiner (29), and a maximum clade credibility (MCC) tree was subsequently generated using TreeAnnotator (29).

10.1128/mBio.01610-20.10 TABLE S5 Viruses included in phylogenetic analyses. Download Table S5, PDF file, 0.1 MB. Copyright © 2020 Su et al. 2020 Su et al. This content is distributed under the terms of the Creative Commons Attribution 4.0 International license.

### Replication kinetics.

Vero-E6 cells were infected with wild-type and Δ382 viruses and fixed at 48 and 72 hpi with 4% paraformaldehyde for 30 min at room temperature. Cells were washed with phosphate-buffered saline (PBS), and SARS-CoV-2 viral antigens were detected using a COVID-19 convalescent human serum at a 1:400 dilution in PBS for 30 min at 37°C. Phycoerythrin (PE)-conjugated goat anti-human IgG polyclonal antibody (eBioscience) was added at a 1:400 dilution and incubated for 30 min at 37°C. For nuclear visualization, cells were stained with 0.01% 4′,6-diamidino-2-phenylindole (DAPI; Abcam). Images were captured by the use of an Eclipse Ti-U fluorescence microscope (Nikon). For virus kinetics analyses, Vero-E6 cells were infected at a multiplicity of infection (MOI) of 0.01. Supernatant was harvested daily for 6 days following infection and 50% tissue culture infective doses (TCID_50_) titers were determined. Statistical analysis was performed using a two-way analysis of variance (ANOVA) with Tukey’s multiple-comparison test. Patient viral load was measured by diagnostic quantitative PCR (qPCR) to determine cycle threshold (*C_T_*) values as previously described (32), and the data were compared using a two-tailed *t* test.

### Subgenomic RNA analysis.

Unambiguous reads which uniquely mapped to the specific joint leader and transcription regulatory sequences (TRS) were counted for each gene from independent NGS library preparations taken from the same RNA extractions of wild-type (*n* = 3) and Δ382 mutant (*n* = 2) SARS-CoV-2 strains collected from two patients. Coding DNA sequence (CDS) and leader and TRS sequence annotations were generated in Geneious and followed published SARS-CoV studies (33). To characterize the differential levels of TRS for each gene, 70 nt of leader sequence and 230 nt downstream of each TRS sequence were annotated individually to form a 300-bp leader-TRS transcript for the splicing-aware aligners in R package rtracklayer (v 1.44). NGS raw fastq reads were then mapped to the reference genome by the use of the Geneious RNA-Seq mapper with the annotation of splice junctions for the leader-TRS. For each gene, the total number of transcripts per million (TPM) of the leader-TRS was calculated in Geneious by excluding ambiguous reads which might have come from other TRS. The resulting TPM data were plotted using ggplot 2 (v3.2.0) in R v3.6.1. Wilcoxon one-sided tests were performed in R to test for significant differences between wild-type and Δ382 samples.

### Data availability.

Sequences generated in this study have been deposited in the GISAID database (see Table S1 for accession numbers). All data are available in the main text or the supplemental material.
