# Supplementary material for: Discovery and Genomic Characterization of a 382-Nucleotide Deletion in ORF7b and ORF8 during the Early Evolution of SARS-CoV-2
Source: mBio. 2020 Jul 21;11(4):e01610-20. doi: 10.1128/mBio.01610-20 (PMC7374062; doi:10.1128/mBio.01610-20)
Supplement: FIG S1 [file mBio.01610-20-sf001.pdf]

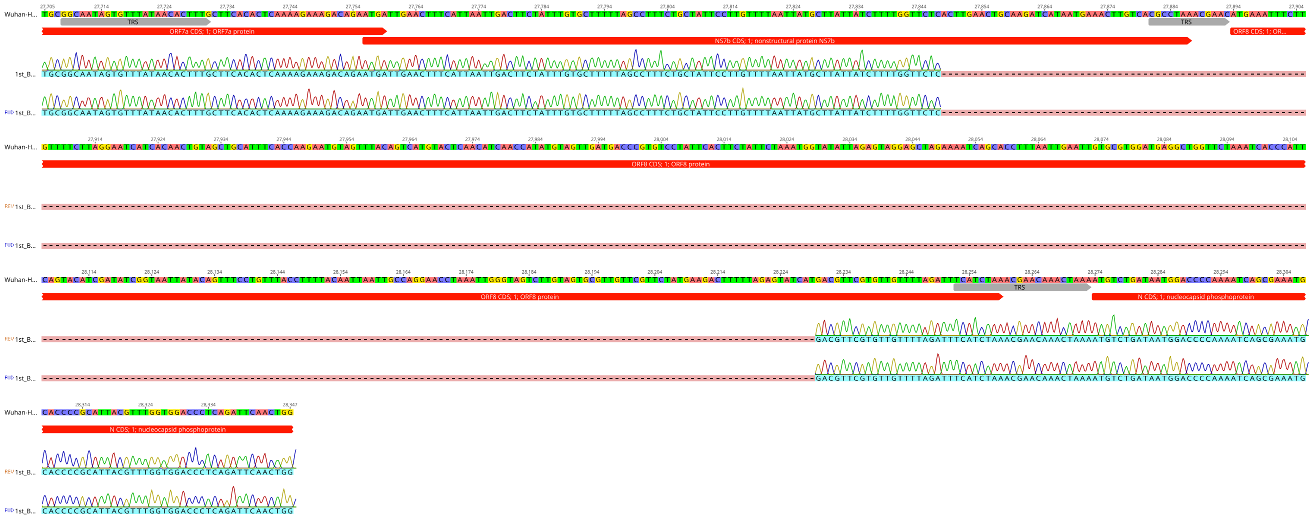

Fig. S1. Sanger sequences of hCoV-19/Singapore/12/2020 Δ382 mapped to Wuhan-Hu-1 showing the position of the 382-nt deletion in the ORF7b and ORF8 regions of the SARS-CoV-2 genome.
