# Supplementary material for: Discovery and Genomic Characterization of a 382-Nucleotide Deletion in ORF7b and ORF8 during the Early Evolution of SARS-CoV-2
Source: mBio. 2020 Jul 21;11(4):e01610-20. doi: 10.1128/mBio.01610-20 (PMC7374062; doi:10.1128/mBio.01610-20)
Supplement: FIG S2 [file mBio.01610-20-sf002.pdf]

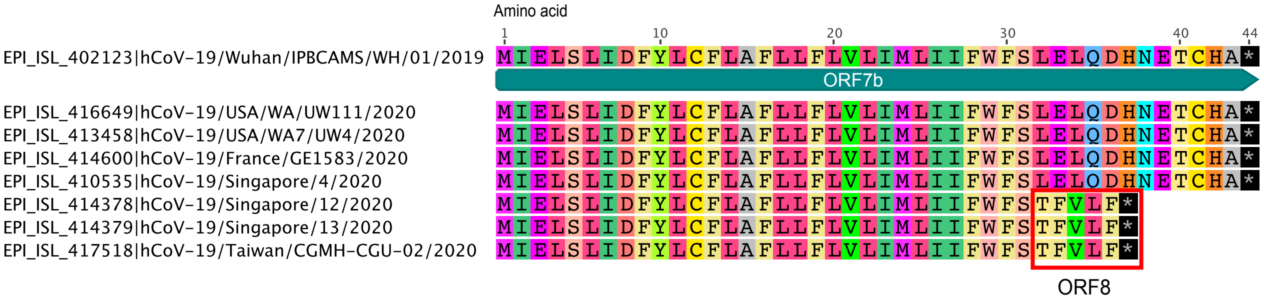

Fig. S2. Amino acid sequences of the ORF7b region in SARS-CoV-2 and SARS-CoV-2 Δ382. Truncated ORF7b is observed as a result of the deleted C terminus of ORF7b in SARS-CoV-2 Δ382.
