# Supplementary material for: Discovery and Genomic Characterization of a 382-Nucleotide Deletion in ORF7b and ORF8 during the Early Evolution of SARS-CoV-2
Source: mBio. 2020 Jul 21;11(4):e01610-20. doi: 10.1128/mBio.01610-20 (PMC7374062; doi:10.1128/mBio.01610-20)
Supplement: TABLE S1 [file mBio.01610-20-st001.docx]

**Table S1.** List of specimens from which full genomes were generated.

| **Accession number** | **Virus name** | **Sample collection date*** | **Deletion** | **Sample type** |
| --- | --- | --- | --- | --- |
| EPI_ISL_407987 | hCoV-19/Singapore/2/2020 | 25-Jan-2020 | Wild type | NP swab (Vero E6) |
| EPI_ISL_407988 | hCoV-19/Singapore/3/2020 | 1-Feb-2020 | Wild type | NP swab (Vero E6) |
| EPI_ISL_410535 | hCoV-19/Singapore/4/2020 | 3-Feb-2020 | Wild type | NP swab (Vero E6) |
| EPI_ISL_410536 | hCoV-19/Singapore/5/2020 | 6-Feb-2020 | Wild type | NP swab (Vero E6) |
| EPI_ISL_410537 | hCoV-19/Singapore/6/2020 | 9-Feb-2020 | Wild type | NP swab (Vero E6) |
| EPI_ISL_414380 | hCoV-19/Singapore/14/2020 | 13-Feb-2020 | 382-nt deletion | NP swab (Vero E6) |
| EPI_ISL_414378 | hCoV-19/Singapore/12/2020 | 17-Feb-2020 | 382-nt deletion | NP swab (Vero E6) |
| EPI_ISL_414379 | hCoV-19/Singapore/13/2020 | 18-Feb-2020 | 382-nt deletion | NP swab (Vero E6) |
| EPI_ISL_420099 | hCoV-19/Singapore/22/2020 | 2-Mar-2020 | 382-nt deletion | NP swab (Vero E6) |
| EPI_ISL_420100 | hCoV-19/Singapore/23/2020 | 2-Mar-2020 | 382-nt deletion | NP swab (Vero E6) |
| EPI_ISL_420101 | hCoV-19/Singapore/24/2020 | 4-Mar-2020 | Wild type | Endotracheal aspirate (Vero E6) |
| EPI_ISL_420102 | hCoV-19/Singapore/25/2020 | 5-Mar-2020 | Wild type | NP swab (Vero E6) |
| EPI_ISL_420103 | hCoV-19/Singapore/26/2020 | 5-Mar-2020 | Wild type | NP swab (Vero E6) |
| EPI_ISL_420104 | hCoV-19/Singapore/27/2020 | 5-Mar-2020 | Wild type | NP swab (Vero E6) |
| EPI_ISL_420105 | hCoV-19/Singapore/28/2020 | 6-Mar-2020 | Wild type | NP swab direct |
| EPI_ISL_420106 | hCoV-19/Singapore/29/2020 | 6-Mar-2020 | Wild type | NP swab direct |
| EPI_ISL_420107 | hCoV-19/Singapore/30/2020 | 9-Mar-2020 | 382-nt deletion | NP swab direct |
| EPI_ISL_420108 | hCoV-19/Singapore/31/2020 | 10-Mar-2020 | Wild type | NP swab direct |
| EPI_ISL_420109 | hCoV-19/Singapore/32/2020 | 11-Mar-2020 | Wild type | NP swab (Vero E6) |
| EPI_ISL_420100 | hCoV-19/Singapore/33/2020 | 11-Mar-2020 | Wild type | NP swab (Vero E6) |
| EPI_ISL_420111 | hCoV-19/Singapore/34/2020 | 12-Mar-2020 | Wild type | Stool direct |

*This date may not correlate to date of diagnosis as patients may have been sampled on multiple days during their illness
