# Supplementary material for: Discovery and Genomic Characterization of a 382-Nucleotide Deletion in ORF7b and ORF8 during the Early Evolution of SARS-CoV-2
Source: mBio. 2020 Jul 21;11(4):e01610-20. doi: 10.1128/mBio.01610-20 (PMC7374062; doi:10.1128/mBio.01610-20)
Supplement: FIG S3 [file mBio.01610-20-sf003.pdf]

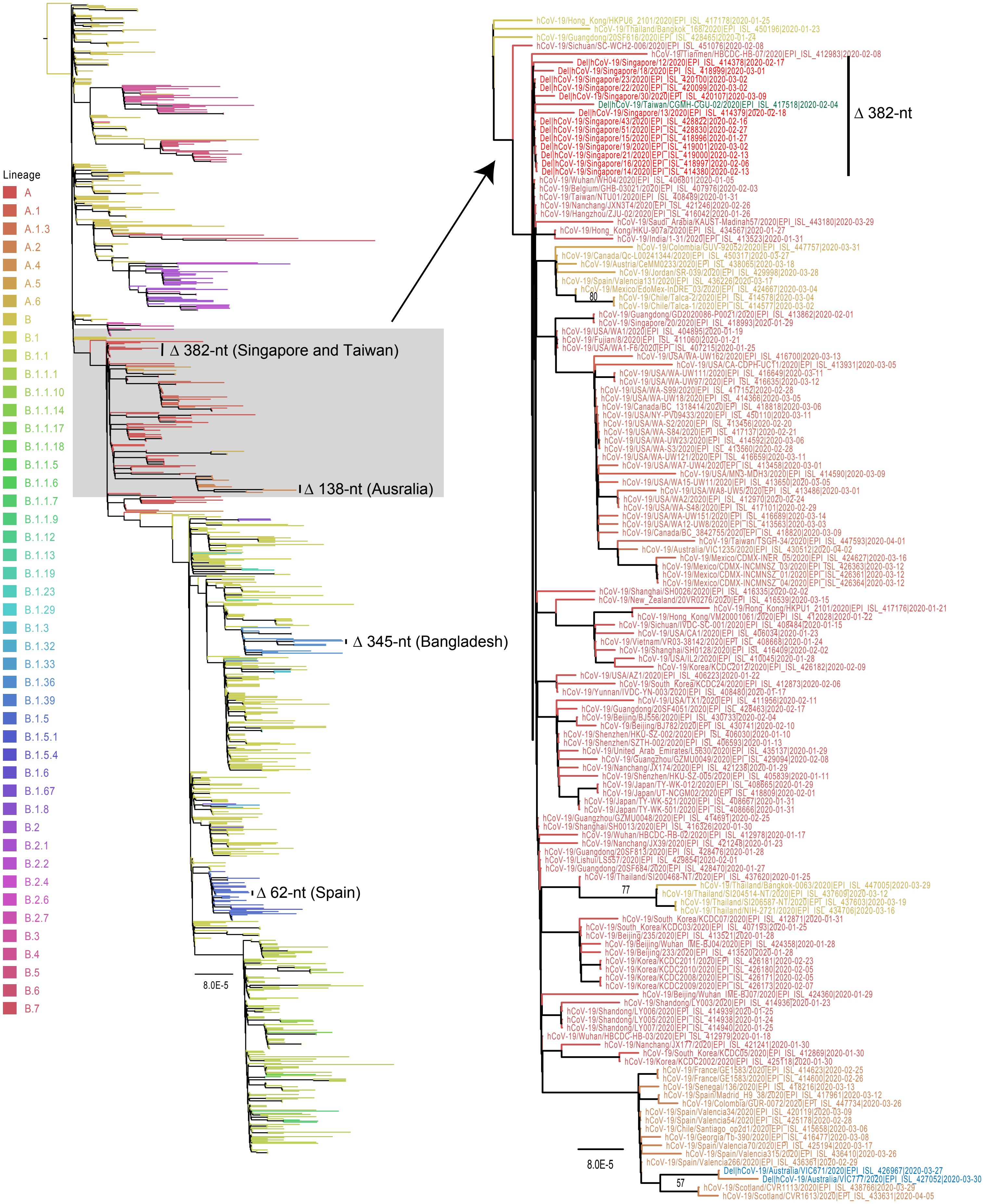

Fig. S3. Maximum-likelihood tree of SARS-CoV-2 genomes (n=1,038) reconstructed using RAxML with bootstrap values >50 indicated at branch nodes.
