# Supplementary material for: Discovery and Genomic Characterization of a 382-Nucleotide Deletion in ORF7b and ORF8 during the Early Evolution of SARS-CoV-2
Source: mBio. 2020 Jul 21;11(4):e01610-20. doi: 10.1128/mBio.01610-20 (PMC7374062; doi:10.1128/mBio.01610-20)
Supplement: FIG S4 [file mBio.01610-20-sf004.pdf]

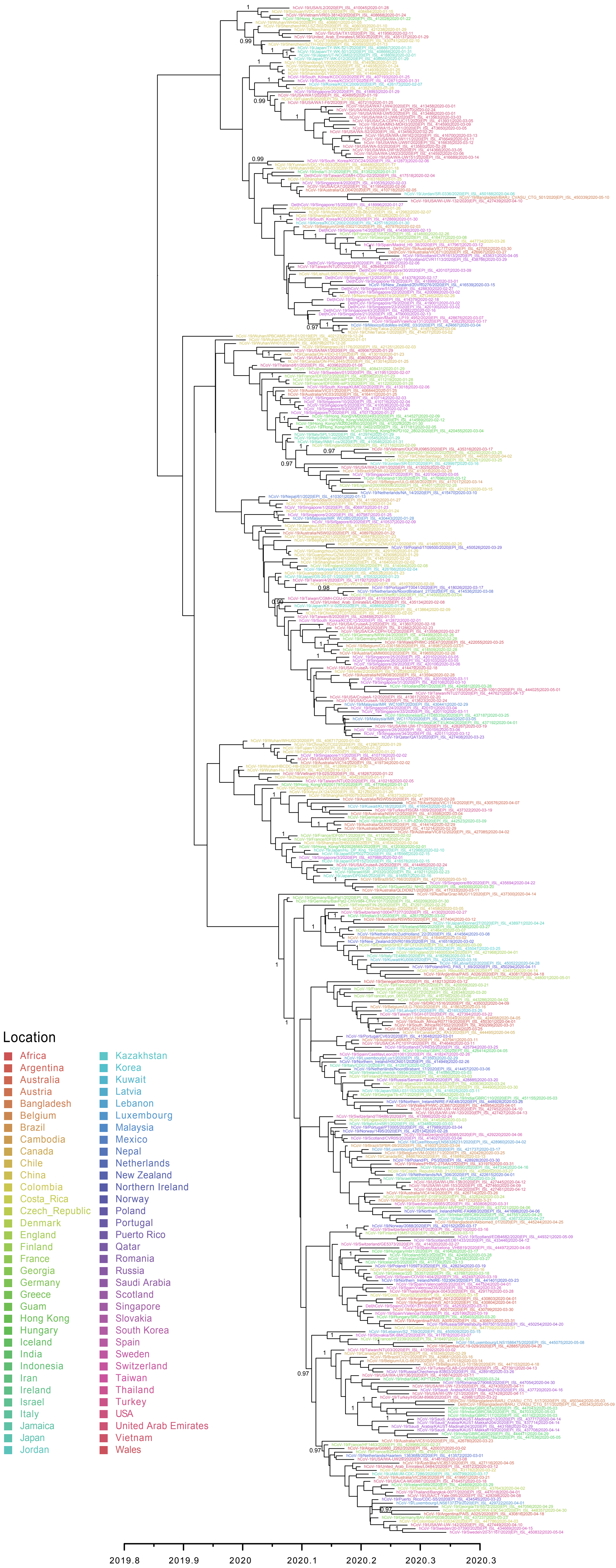

Fig. S4. Evolutionary relationships of human SARS-CoV-2 and SARS-CoV-2 Δ382. Temporal phylogeny of 419 complete genomes inferred using an uncorrelated lognormal relaxed clock model in BEAST. Coloured virus names represent different geographic locations. Bayesian posterior probabilities of ≥0.95 are indicated at nodes. Scale bar represents time in years.
