# Supplementary material for: Discovery and Genomic Characterization of a 382-Nucleotide Deletion in ORF7b and ORF8 during the Early Evolution of SARS-CoV-2
Source: mBio. 2020 Jul 21;11(4):e01610-20. doi: 10.1128/mBio.01610-20 (PMC7374062; doi:10.1128/mBio.01610-20)
Supplement: TABLE S2 [file mBio.01610-20-st002.docx]

**Table S2.** Comparison of genome similarity between SARS-CoV-2 ∆382 viruses with the wild-type virus Wuhan-Hu-1 SARS-CoV-2.

|  | 382-nt deletion variants | |
| --- | --- | --- |
|  | Length | % nt identity |
| orf1ab | 21,291 | 99.8 |
| S | 3,822 | 99.2–100 |
| ORF3a | 828 | 99.9-100 |
| E | 228 | 100 |
| M | 669 | 100 |
| ORF6 | 186 | 100 |
| ORF7a | 366 | 100 |
| ORF7b | 132 | 100 |
| ORF8* | 366 | 100 |
| N | 1,260 | 99.9–100 |
| ORF10 | 117 | 100 |

*Comparison was made excluding the area of the 382-nt deletion
