# Supplementary material for: Discovery and Genomic Characterization of a 382-Nucleotide Deletion in ORF7b and ORF8 during the Early Evolution of SARS-CoV-2
Source: mBio. 2020 Jul 21;11(4):e01610-20. doi: 10.1128/mBio.01610-20 (PMC7374062; doi:10.1128/mBio.01610-20)
Supplement: TABLE S3 [file mBio.01610-20-st003.docx]

**Table S3.** Estimated nodes dates within the SARS-CoV-2 phylogeny shown in Fig. 1C.

| **Node** | **Mean TMRCA** | **Lower 95% HPD** | **Upper 95% HPD** |
| --- | --- | --- | --- |
| Root age | 2019.86 (11 Nov 2019) | 2019.76 (06 Oct 2019) | 2019.92 (03 Dec 2019) |
| A | 2020.96 (17 Dec 2020) | 2019.90 (26 Nov 2019) | 2020.00 (01 Jan 2020) |
| B | 2020.02 (09 Jan 2020) | 2019.98 (27 Dec 2019) | 2020.04 (16 Jan 2020) |

Abbreviations: TMRCA, time to most recent common ancestor, HPD: highest posterior density.
