# Supplementary material for: Discovery and Genomic Characterization of a 382-Nucleotide Deletion in ORF7b and ORF8 during the Early Evolution of SARS-CoV-2
Source: mBio. 2020 Jul 21;11(4):e01610-20. doi: 10.1128/mBio.01610-20 (PMC7374062; doi:10.1128/mBio.01610-20)
Supplement: TABLE S4 [file mBio.01610-20-st004.docx]

**Table S4.** Summary of deletion variants of SARS-CoV-2.

| **Deletion variants** | **Deletions** | **Deleted position** | **Lineage** |
| --- | --- | --- | --- |
| hCoV-19/Singapore/12/2020\|EPI_ISL_414378\|2020-02-17 | 382-nt | 27848–28229 | A |
| hCoV-19/Singapore/13/2020\|EPI_ISL_414379\|2020-02-18 | 382-nt | 27848–28229 | A |
| hCoV-19/Singapore/14/2020\|EPI_ISL_414380\|2020-02-13 | 382-nt | 27848–28229 | A |
| hCoV-19/Singapore/19/2020\|EPI_ISL_419001\|2020-03-02 | 382-nt | 27848–28229 | A |
| hCoV-19/Singapore/21/2020\|EPI_ISL_419000\|2020-02-13 | 382-nt | 27848–28229 | A |
| hCoV-19/Singapore/22/2020\|EPI_ISL_420099\|2020-03-02 | 382-nt | 27848–28229 | A |
| hCoV-19/Singapore/23/2020\|EPI_ISL_420100\|2020-03-02 | 382-nt | 27848–28229 | A |
| hCoV-19/Singapore/51/2020\|EPI_ISL_428830\|2020-02-27 | 382-nt | 27848–28229 | A |
| hCoV-19/Singapore/15/2020\|EPI_ISL_418996\|2020-01-27 | 382-nt | 27848–28229 | A |
| hCoV-19/Singapore/16/2020\|EPI_ISL_418997\|2020-02-06 | 382-nt | 27848–28229 | A |
| hCoV-19/Singapore/18/2020\|EPI_ISL_418999\|2020-03-01 | 382-nt | 27848–28229 | A |
| hCoV-19/Singapore/30/2020\|EPI_ISL_420107\|2020-03-09 | 382-nt | 27848–28229 | A |
| hCoV-19/Singapore/43/2020\|EPI_ISL_428822\|2020-02-16 | 382-nt | 27848–28229 | A |
| hCoV-19/Taiwan/CGMH-CGU-02/2020\|EPI_ISL_417518\|2020-02-04 | 382-nt | 27848–28229 | A |
| hCoV-19/Australia/VIC671/2020\|EPI_ISL_426967\|2020-03-27 | 138-nt | 27846–27983 | A.2 |
| hCoV-19/Australia/VIC777/2020\|EPI_ISL_427052\|2020-03-30 | 138-nt | 27846–27983 | A.2 |
| hCoV-19/Spain/COV001371/2020\|EPI_ISL_452530\|2020-03-13 | 62-nt | 27904–27965 | B.1.5 |
| hCoV-19/Spain/COV001404/2020\|EPI_ISL_452497\|2020-03-19 | 62-nt | 27904–27965 | B.1.5 |
| hCoV-19/Bangladesh/BARJ_CVASU_CTG_511/2020\|EPI_ISL_450343\|2020-05-09 | 345-nt | 27910–28254 | B.1.36 |
| hCoV-19/Bangladesh/BARJ_CVASU_CTG_517/2020\|EPI_ISL_450344\|2020-05-03 | 345-nt | 27910–28254 | B1.3.6 |
