# Supplementary material for: Discovery and Genomic Characterization of a 382-Nucleotide Deletion in ORF7b and ORF8 during the Early Evolution of SARS-CoV-2
Source: mBio. 2020 Jul 21;11(4):e01610-20. doi: 10.1128/mBio.01610-20 (PMC7374062; doi:10.1128/mBio.01610-20)
Supplement: FIG S5 [file mBio.01610-20-sf005.pdf]

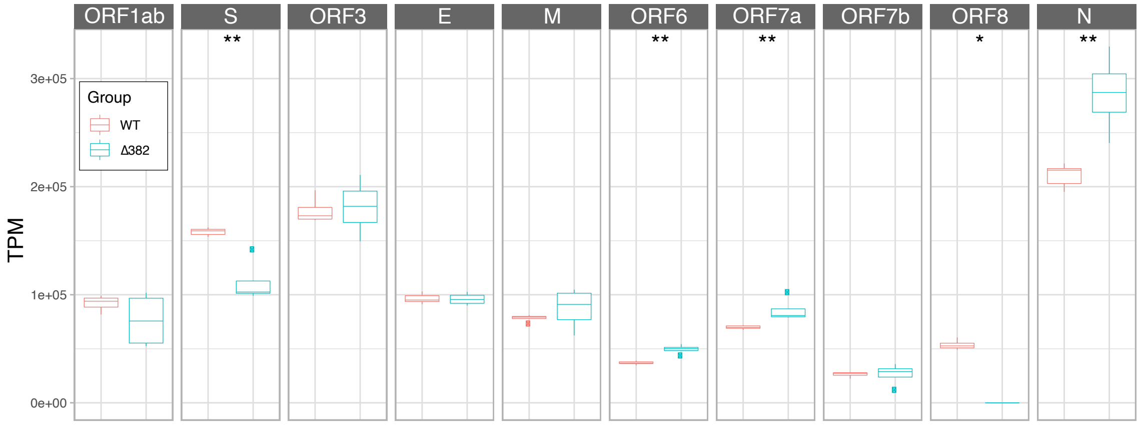

Fig. S5. Comparison of transcription of different SARS-CoV-2 genes in wild-type (WT) versus Δ382 viruses. Abundance of mapped reads relative to transcriptional regulatory sequence (TRS) positions across the genome. Transcripts per million (TPM) reads were calculated from reads mapped specifically to each leader-TRS region and a whisker and scatter plot was drawn for each gene. A Wilcoxon test was applied to the TPM for each gene of Δ382 to WT (*: $p \leq 0.05$ **: $p \leq 0.01$).
