# Supplementary material for: Discovery and Genomic Characterization of a 382-Nucleotide Deletion in ORF7b and ORF8 during the Early Evolution of SARS-CoV-2
Source: mBio. 2020 Jul 21;11(4):e01610-20. doi: 10.1128/mBio.01610-20 (PMC7374062; doi:10.1128/mBio.01610-20)
Supplement: TABLE S5 [file mBio.01610-20-st005.pdf]

Table S5. Viruses included in phylogenetic analyses.

Accession number	Virus isolate	Collection date	Note
EPI_ISL_407987	hCoV_19/Singapore/2/2020	2020-01-25	Wild type (this study)
EPI_ISL_407988	hCoV_19/Singapore/3/2020	2020-02-01	Wild type (this study)
EPI_ISL_410535	hCoV_19/Singapore/4/2020	2020-02-03	Wild type (this study)
EPI_ISL_410536	hCoV-19/Singapore/5/2020	2020-02-06	Wild type (this study)
EPI_ISL_410537	hCoV_19/Singapore/6/2020	2020-02-09	Wild type (this study)
EPI_ISL_414378	hCoV-19/Singapore/12/2020	2020-02-17	382-nt deletion (this study)
EPI_ISL_414379	hCoV-19/Singapore/13/2020	2020-02-18	382-nt deletion (this study)
EPI_ISL_414380	hCoV-19/Singapore/14/2020	2020-02-13	382-nt deletion (this study)
EPI_ISL_420099	hCoV-19/Singapore/22/2020	2020-03-02	382-nt deletion (this study)
EPI_ISL_420100	hCoV-19/Singapore/23/2020	2020-03-02	382-nt deletion (this study)
EPI_ISL_420101	hCoV_19/Singapore/24/2020	2020-03-04	Wild type (this study)
EPI_ISL_420102	hCoV_19/Singapore/25/2020	2020-03-05	Wild type (this study)
EPI_ISL_420103	hCoV_19/Singapore/26/2020	2020-03-05	Wild type (this study)
EPI_ISL_420104	hCoV_19/Singapore/27/2020	2020-03-05	Wild type (this study)
EPI_ISL_420105	hCoV_19/Singapore/28/2020	2020-03-06	Wild type (this study)
EPI_ISL_420106	hCoV_19/Singapore/29/2020	2020-03-06	Wild type (this study)
EPI_ISL_420107	hCoV-19/Singapore/30/2020	2020-03-09	382-nt deletion (this study)
EPI_ISL_420108	hCoV-19/Singapore/31/2020	2020-03-10	Wild type (this study)
EPI_ISL_420109	hCoV_19/Singapore/32/2020	2020-03-11	Wild type (this study)
EPI_ISL_420110	hCoV_19/Singapore/33/2020	2020-03-11	Wild type (this study)
EPI_ISL_420111	hCoV_19/Singapore/34/2020	2020-03-12	Wild type (this study)
EPI_ISL_402120	hCoV-19/Wuhan/IVDC-HB-04/2020	2020-01-01	Wild type
EPI_ISL_402123	hCoV-19/Wuhan/IPBCAMS-WH-01/2019	2019-12-24	Wild type
EPI_ISL_402125	hCoV-19/Wuhan-Hu-1/2019	2019-12-31	Wild type
EPI_ISL_403962	hCoV-19/Thailand/61/2020	2020-01-08	Wild type
EPI_ISL_404228	hCoV-19/Zhejiang/WZ-02/2020	2020-01-17	Wild type
EPI_ISL_404895	hCoV-19/USA/WA1/2020	2020-01-19	Wild type
EPI_ISL_406030	hCoV-19/Shenzhen/HKU-SZ-002/2020	2020-01-10	Wild type
EPI_ISL_406536	hCoV-19/Foshan/20SF211/2020	2020-01-22	Wild type
EPI_ISL_406538	hCoV-19/Guangdong/20SF201/2020	2020-01-23	Wild type
EPI_ISL_406593	hCoV-19/Shenzhen/SZTH-002/2020	2020-01-13	Wild type
EPI_ISL_406596	hCoV-19/France/IDF0372/2020	2020-01-23	Wild type
EPI_ISL_406717	hCoV-19/Wuhan/WHU02/2020	2020-01-02	Wild type
EPI_ISL_406798	hCoV-19/Wuhan/WH01/2019	2019-12-26	Wild type
EPI_ISL_406801	hCoV-19/Wuhan/WH04/2020	2020-01-05	Wild type
EPI_ISL_406844	hCoV-19/Australia/VIC01/2020	2020-01-25	Wild type
EPI_ISL_406862	hCoV-19/Germany/BavPat1/2020	2020-01-28	Wild type
EPI_ISL_406973	hCoV_19/Singapore/1/2020	2020-01-23	Wild type
EPI_ISL_407193	hCoV-19/South_Korea/KCDC03/2020	2020-01-25	Wild type
EPI_ISL_407215	hCoV-19/USA/WA1-F6/2020	2020-01-25	Wild type
EPI_ISL_407976	hCoV-19/Belgium/GHB-03021/2020	2020-02-03	Wild type
EPI_ISL_408008	hCoV-19/USA/CA3/2020	2020-01-29	Wild type
EPI_ISL_408431	hCoV-19/France/IDF0626/2020	2020-01-29	Wild type
EPI_ISL_408479	hCoV-19/Chongqing/ZX01/2020	2020-01-23	Wild type
EPI_ISL_408480	hCoV-19/Yunnan/IVDC-YN-003/2020	2020-01-17	Wild type
EPI_ISL_408481	hCoV-19/Chongqing/IVDC-CQ-001/2020	2020-01-18	Wild type
EPI_ISL_408484	hCoV-19/Sichuan/IVDC-SC-001/2020	2020-01-15	Wild type
EPI_ISL_408489	hCoV-19/Taiwan/NTU01/2020	2020-01-31	Wild type
EPI_ISL_408665	hCoV-19/Japan/TY-WK-012/2020	2020-01-29	Wild type
EPI_ISL_408666	hCoV-19/Japan/TY-WK-501/2020	2020-01-31	Wild type
EPI_ISL_408667	hCoV-19/Japan/TY-WK-521/2020	2020-01-31	Wild type
EPI_ISL_408668	hCoV-19/Vietnam/VR03-38142/2020	2020-01-24	Wild type
EPI_ISL_408669	hCoV-19/Japan/KY-V-029/2020	2020-01-29	Wild type
EPI_ISL_408670	hCoV-19/USA/WI1/2020	2020-01-31	Wild type
EPI_ISL_408976	hCoV-19/Australia/NSW02/2020	2020-01-22	Wild type

EPI_ISL_409067	hCoV-19/USA/MA1/2020	2020-01-29	Wild type
EPI_ISL_410045	hCoV-19/USA/IL2/2020	2020-01-28	Wild type
EPI_ISL_410218	hCoV-19/Taiwan/NTU02/2020	2020-02-05	Wild type
EPI_ISL_410301	hCoV-19/Nepal/61/2020	2020-01-13	Wild type
EPI_ISL_410532	hCoV-19/Japan/OS-20-07-1/2020	2020-01-23	Wild type
EPI_ISL_410545	hCoV-19/Italy/INMI1-isl/2020	2020-01-29	Wild type
EPI_ISL_410546	hCoV-19/Italy/INMI1-cs/2020	2020-01-31	Wild type
EPI_ISL_410713	hCoV_19/Singapore/7/2020	2020-01-27	Wild type
EPI_ISL_410714	hCoV_19/Singapore/8/2020	2020-02-03	Wild type
EPI_ISL_410715	hCoV_19/Singapore/9/2020	2020-02-04	Wild type
EPI_ISL_410716	hCoV_19/Singapore/10/2020	2020-02-04	Wild type
EPI_ISL_410718	hCoV-19/Australia/QLD04/2020	2020-02-05	Wild type
EPI_ISL_410719	hCoV_19/Singapore/11/2020	2020-02-02	Wild type
EPI_ISL_410984	hCoV-19/France/IDF0515-isl/2020	2020-01-29	Wild type
EPI_ISL_411060	hCoV-19/Fujian/8/2020	2020-01-21	Wild type
EPI_ISL_411066	hCoV-19/Fujian/13/2020	2020-01-22	Wild type
EPI_ISL_411218	hCoV-19/France/IDF0571/2020	2020-02-02	Wild type
EPI_ISL_411219	hCoV-19/France/IDF0386-islP1/2020	2020-01-28	Wild type
EPI_ISL_411220	hCoV-19/France/IDF0386-islP3/2020	2020-01-28	Wild type
EPI_ISL_411902	hCoV-19/Cambodia/0012/2020	2020-01-27	Wild type
EPI_ISL_411915	hCoV-19/Taiwan/CGMH-CGU-01/2020	2020-01-25	Wild type
EPI_ISL_411927	hCoV-19/Taiwan/4/2020	2020-01-28	Wild type
EPI_ISL_411950	hCoV-19/Jiangsu/JS01/2020	2020-01-23	Wild type
EPI_ISL_411951	hCoV-19/Sweden/01/2020	2020-02-07	Wild type
EPI_ISL_411953	hCoV-19/Jiangsu/JS03/2020	2020-01-24	Wild type
EPI_ISL_411954	hCoV-19/USA/CA7/2020	2020-02-06	Wild type
EPI_ISL_411956	hCoV-19/USA/TX1/2020	2020-02-11	Wild type
EPI_ISL_412026	hCoV-19/Hefei/2/2020	2020-02-23	Wild type
EPI_ISL_412028	hCoV-19/Hong_Kong/VM20001061/2020	2020-01-22	Wild type
EPI_ISL_412029	hCoV-19/Hong_Kong/VB20024950/2020	2020-01-30	Wild type
EPI_ISL_412030	hCoV-19/Hong_Kong/VB20026565/2020	2020-02-01	Wild type
EPI_ISL_412116	hCoV-19/England/09c/2020	2020-02-09	Wild type
EPI_ISL_412862	hCoV-19/USA/CA9/2020	2020-02-23	Wild type
EPI_ISL_412869	hCoV-19/South_Korea/KCDC05/2020	2020-01-30	Wild type
EPI_ISL_412871	hCoV-19/South_Korea/KCDC07/2020	2020-01-31	Wild type
EPI_ISL_412872	hCoV-19/South_Korea/KCDC12/2020	2020-02-01	Wild type
EPI_ISL_412873	hCoV-19/South_Korea/KCDC24/2020	2020-02-06	Wild type
EPI_ISL_412899	hCoV-19/Wuhan/HBCDC-HB-03/2019	2019-12-30	Wild type
EPI_ISL_412966	hCoV-19/China/IQTC01/2020	2020-02-05	Wild type
EPI_ISL_412967	hCoV-19/China/IQTC02/2020	2020-01-29	Wild type
EPI_ISL_412968	hCoV-19/Japan/Hu_DP_Kng_19-020/2020	2020-02-10	Wild type
EPI_ISL_412970	hCoV-19/USA/WA2/2020	2020-02-24	Wild type
EPI_ISL_412971	hCoV-19/Finland/FIN-25/2020	2020-02-25	Wild type
EPI_ISL_412973	hCoV-19/Italy/CDG1/2020	2020-02-20	Wild type
EPI_ISL_412974	hCoV-19/Italy/SPL1/2020	2020-01-29	Wild type
EPI_ISL_412975	hCoV-19/Australia/NSW05/2020	2020-02-28	Wild type
EPI_ISL_412979	hCoV-19/Wuhan/HBCDC-HB-03/2020	2020-01-18	Wild type
EPI_ISL_412982	hCoV-19/Wuhan/HBCDC-HB-06/2020	2020-02-07	Wild type
EPI_ISL_413014	hCoV-19/Canada/ON-PHL2445/2020	2020-01-25	Wild type
EPI_ISL_413015	hCoV-19/Canada/ON-VIDO-01/2020	2020-01-23	Wild type
EPI_ISL_413016	hCoV-19/Brazil/SPBR-02/2020	2020-02-28	Wild type
EPI_ISL_413018	hCoV-19/South_Korea/KUMC02/2020	2020-02-06	Wild type
EPI_ISL_413020	hCoV-19/Switzerland/1000477377/2020	2020-02-27	Wild type
EPI_ISL_413025	hCoV-19/USA/WA3-UW1/2020	2020-02-27	Wild type
EPI_ISL_413214	hCoV-19/Australia/NSW07/2020	2020-02-29	Wild type
EPI_ISL_413456	hCoV-19/USA/WA-S2/2020	2020-02-20	Wild type
EPI_ISL_413458	hCoV-19/USA/WA7-UW4/2020	2020-03-01	Wild type
EPI_ISL_413459	hCoV-19/Japan/TK-20-31-3/2020	2020-02-20	Wild type

EPI_ISL_413486	hCoV-19/USA/WA8-UW5/2020	2020-03-01	Wild type
EPI_ISL_413488	hCoV-19/Germany/NRW-01/2020	2020-02-28	Wild type
EPI_ISL_413489	hCoV-19/Italy/UniSR1/2020	2020-03-03	Wild type
EPI_ISL_413521	hCoV-19/Beijing/235/2020	2020-01-28	Wild type
EPI_ISL_413523	hCoV-19/India/1-31/2020	2020-01-31	Wild type
EPI_ISL_413558	hCoV-19/USA/CA-CDPH-UC2/2020	2020-02-27	Wild type
EPI_ISL_413560	hCoV-19/USA/WA-S3/2020	2020-02-28	Wild type
EPI_ISL_413563	hCoV-19/USA/WA12-UW8/2020	2020-03-03	Wild type
EPI_ISL_413572	hCoV-19/Netherlands/Haarlem_1363688/2020	2020-03-01	Wild type
EPI_ISL_413592	hCoV-19/Taiwan/NTU03/2020	2020-03-02	Wild type
EPI_ISL_413593	hCoV-19/Luxembourg/Lux1/2020	2020-02-29	Wild type
EPI_ISL_413594	hCoV-19/Australia/NSW08/2020	2020-02-28	Wild type
EPI_ISL_413598	hCoV-19/Australia/NSW12/2020	2020-03-04	Wild type
EPI_ISL_413602	hCoV-19/Finland/FIN03032020A/2020	2020-03-03	Wild type
EPI_ISL_413607	hCoV-19/USA/CruiseA-2/2020	2020-02-18	Wild type
EPI_ISL_413617	hCoV-19/USA/CruiseA-12/2020	2020-02-20	Wild type
EPI_ISL_413623	hCoV-19/USA/CruiseA-18/2020	2020-02-24	Wild type
EPI_ISL_413648	hCoV-19/Portugal/CV63/2020	2020-03-01	Wild type
EPI_ISL_413650	hCoV-19/USA/WA15-UW11/2020	2020-03-05	Wild type
EPI_ISL_413864	hCoV-19/Guangdong/GD2020246-P0028/2020	2020-02-09	Wild type
EPI_ISL_413931	hCoV-19/USA/CA-CDPH-UC11/2020	2020-03-05	Wild type
EPI_ISL_413996	hCoV-19/Switzerland/TI9486/2020	2020-02-24	Wild type
EPI_ISL_414011	hCoV-19/England/200990006/2020	2020-02-26	Wild type
EPI_ISL_414020	hCoV-19/Switzerland/GE5373/2020	2020-02-27	Wild type
EPI_ISL_414027	hCoV-19/Scotland/CVR05/2020	2020-03-04	Wild type
EPI_ISL_414044	hCoV-19/England/200690756/2020	2020-02-08	Wild type
EPI_ISL_414366	hCoV-19/USA/WA-UW18/2020	2020-03-05	Wild type
EPI_ISL_414414	hCoV-19/Australia/QLD09/2020	2020-02-29	Wild type
EPI_ISL_414457	hCoV-19/Netherlands/NoordBrabant_17/2020	2020-03-06	Wild type
EPI_ISL_414479	hCoV-19/USA/CruiseA-19/2020	2020-02-18	Wild type
EPI_ISL_414485	hCoV-19/USA/CruiseA-26/2020	2020-02-24	Wild type
EPI_ISL_414499	hCoV-19/Germany/NRW-04/2020	2020-02-26	Wild type
EPI_ISL_414500	hCoV-19/England/Sheff01/2020	2020-03-04	Wild type
EPI_ISL_414509	hCoV-19/Germany/NRW-09/2020	2020-02-28	Wild type
EPI_ISL_414510	hCoV-19/Shanghai/SH01/2020	2020-02-02	Wild type
EPI_ISL_414520	hCoV-19/Germany/BavPat2/2020	2020-03-02	Wild type
EPI_ISL_414526	hCoV-19/England/201040141/2020	2020-03-03	Wild type
EPI_ISL_414527	hCoV-19/Hong_Kong/VM20002493/2020	2020-02-09	Wild type
EPI_ISL_414536	hCoV-19/Netherlands/NoordBrabant_27/2020	2020-03-08	Wild type
EPI_ISL_414564	hCoV-19/Netherlands/ZuidHolland_22/2020	2020-03-08	Wild type
EPI_ISL_414569	hCoV-19/Hong_Kong/VM20002582/2020	2020-02-12	Wild type
EPI_ISL_414577	hCoV-19/Chile/Talca-1/2020	2020-03-02	Wild type
EPI_ISL_414578	hCoV-19/Chile/Talca-2/2020	2020-03-04	Wild type
EPI_ISL_414580	hCoV-19/Chile/Santiago-2/2020	2020-03-05	Wild type
EPI_ISL_414586	hCoV-19/Ireland/Limerick-19934/2020	2020-03-03	Wild type
EPI_ISL_414590	hCoV-19/USA/MN3-MDH3/2020	2020-03-09	Wild type
EPI_ISL_414592	hCoV-19/USA/WA-UW23/2020	2020-03-06	Wild type
EPI_ISL_414600	hCoV-19/France/GE1583/2020	2020-02-26	Wild type
EPI_ISL_414616	hCoV-19/USA/WA-UW29/2020	2020-03-08	Wild type
EPI_ISL_414643	hCoV-19/Finland/FIN-508/2020	2020-03-07	Wild type
EPI_ISL_414648	hCoV-19/USA/CA-PC101P/2020	2020-03-11	Wild type
EPI_ISL_414687	hCoV-19/Guangzhou/GZMU0031/2020	2020-02-25	Wild type
EPI_ISL_414936	hCoV-19/Shandong/LY003/2020	2020-01-23	Wild type
EPI_ISL_414938	hCoV-19/Shandong/LY005/2020	2020-01-24	Wild type
EPI_ISL_414939	hCoV-19/Shandong/LY006/2020	2020-01-25	Wild type
EPI_ISL_414940	hCoV-19/Shandong/LY007/2020	2020-01-25	Wild type
EPI_ISL_414949	hCoV-19/Northern_Ireland/HSCNI01/2020	2020-02-26	Wild type
EPI_ISL_415470	hCoV-19/Netherlands/NA_14/2020	2020-03-10	Wild type

EPI_ISL_415642	hCoV-19/Georgia/Tb-477/2020	2020-03-10	Wild type
EPI_ISL_416031	hCoV-19/Brazil/SPBR-09/2020	2020-03-04	Wild type
EPI_ISL_416316	hCoV-19/Shanghai/SH0002/2020	2020-01-25	Wild type
EPI_ISL_416326	hCoV-19/Shanghai/SH0013/2020	2020-01-30	Wild type
EPI_ISL_416342	hCoV-19/Shanghai/SH0033/2020	2020-02-04	Wild type
EPI_ISL_416373	hCoV-19/Shanghai/SH0070/2020	2020-02-07	Wild type
EPI_ISL_416405	hCoV-19/Shanghai/SH0121/2020	2020-02-02	Wild type
EPI_ISL_416411	hCoV-19/Australia/VIC03/2020	2020-01-25	Wild type
EPI_ISL_416426	hCoV-19/Hungary/mbl1/2020	2020-03-17	Wild type
EPI_ISL_416457	hCoV-19/USA/CA-MG0987/2020	2020-03-18	Wild type
EPI_ISL_416468	hCoV-19/Belgium/GMH-03022/2020	2020-03-02	Wild type
EPI_ISL_416477	hCoV-19/Georgia/Tb-390/2020	2020-03-08	Wild type
EPI_ISL_416497	hCoV-19/France/HF2239/2020	2020-03-10	Wild type
EPI_ISL_416511	hCoV-19/France/B2348/2020	2020-03-07	Wild type
EPI_ISL_416519	hCoV-19/New_Zealand/20VR0189/2020	2020-03-02	Wild type
EPI_ISL_416525	hCoV-19/Japan/SMU-0311S3/2020	2020-03-11	Wild type
EPI_ISL_416539	hCoV-19/New_Zealand/20VR0276/2020	2020-03-15	Wild type
EPI_ISL_416543	hCoV-19/Kuwait/KU18/2020	2020-03-02	Wild type
EPI_ISL_416566	hCoV-19/Japan/DP0027/2020	2020-02-15	Wild type
EPI_ISL_416578	hCoV-19/Japan/DP0152/2020	2020-02-15	Wild type
EPI_ISL_416597	hCoV-19/Japan/DP0346/2020	2020-02-16	Wild type
EPI_ISL_416635	hCoV-19/USA/WA-UW97/2020	2020-03-12	Wild type
EPI_ISL_416649	hCoV-19/USA/WA-UW111/2020	2020-03-11	Wild type
EPI_ISL_416674	hCoV-19/USA/WA-UW136/2020	2020-03-11	Wild type
EPI_ISL_416689	hCoV-19/USA/WA-UW151/2020	2020-03-14	Wild type
EPI_ISL_416700	hCoV-19/USA/WA-UW162/2020	2020-03-13	Wild type
EPI_ISL_416734	hCoV-19/England/SHEF-BFCFD/2020	2020-03-09	Wild type
EPI_ISL_416750	hCoV-19/France/Lyon_683/2020	2020-03-06	Wild type
EPI_ISL_416756	hCoV-19/France/Lyon_06531/2020	2020-03-06	Wild type
EPI_ISL_417017	hCoV-19/Belgium/ULG-6638/2020	2020-03-14	Wild type
EPI_ISL_417018	hCoV-19/Belgium/ULG-6670/2020	2020-03-14	Wild type
EPI_ISL_417033	hCoV-19/Australia/QLDID921/2020	2020-03-11	Wild type
EPI_ISL_417064	hCoV-19/Hong_Kong/VB20017970/2020	2020-01-21	Wild type
EPI_ISL_417181	hCoV-19/Hong_Kong/HKPU19_0402/2020	2020-02-05	Wild type
EPI_ISL_417404	hCoV-19/Australia/NSW50/2020	2020-03-12	Wild type
EPI_ISL_417518	hCoV-19/Taiwan/CGMH-CGU-02/2020	2020-02-04	382-nt deletion
EPI_ISL_417696	hCoV-19/Iceland/135/2020	2020-03-12	Wild type
EPI_ISL_417739	hCoV-19/Iceland/5/2020	2020-03-13	Wild type
EPI_ISL_417878	hCoV-19/Slovakia/SK-BMC2/2020	2020-03-07	Wild type
EPI_ISL_417961	hCoV-19/Spain/Madrid_H9_38/2020	2020-03-12	Wild type
EPI_ISL_417989	hCoV-19/Portugal/PT0005/2020	2020-03-04	Wild type
EPI_ISL_418026	hCoV-19/Portugal/PT0041/2020	2020-03-17	Wild type
EPI_ISL_418213	hCoV-19/Senegal/094/2020	2020-03-12	Wild type
EPI_ISL_418247	hCoV-19/Spain/CastillayLeon201061/2020	2020-02-26	Wild type
EPI_ISL_418256	hCoV-19/Italy/TE4880/2020	2020-03-14	Wild type
EPI_ISL_418267	hCoV-19/Vietnam/19-02S/2020	2020-01-22	Wild type
EPI_ISL_418345	hCoV-19/Canada/ON_PHL8751/2020	2020-02-29	Wild type
EPI_ISL_418391	hCoV-19/Finland/13M57/2020	2020-03-13	Wild type
EPI_ISL_418511	hCoV-19/Hangzhou/HZ477/2020	2020-01-24	Wild type
EPI_ISL_418632	hCoV-19/Belgium/ULG-7500/2020	2020-03-16	Wild type
EPI_ISL_418809	hCoV-19/Japan/UT-NCGM02/2020	2020-02-01	Wild type
EPI_ISL_418849	hCoV-19/Canada/BC_8486790/2020	2020-03-13	Wild type
EPI_ISL_418987	hCoV-19/Belgium/CG-030158/2020	2020-03-01	Wild type
EPI_ISL_418993	hCoV-19/Singapore/20/2020	2020-01-29	Wild type
EPI_ISL_418996	hCoV-19/Singapore/15/2020	2020-01-27	382-nt deletion
EPI_ISL_418997	hCoV-19/Singapore/16/2020	2020-02-06	382-nt deletion
EPI_ISL_418999	hCoV-19/Singapore/18/2020	2020-03-01	382-nt deletion
EPI_ISL_419000	hCoV-19/Singapore/21/2020	2020-02-13	382-nt deletion

EPI_ISL_419001	hCoV-19/Singapore/19/2020	2020-03-02	382-nt deletion
EPI_ISL_419211	hCoV-19/Israel/ISR_JP0320/2020	2020-02-23	Wild type
EPI_ISL_419655	hCoV-19/Austria/CeMM0002/2020	2020-02-26	Wild type
EPI_ISL_419734	hCoV-19/Australia/VIC14/2020	2020-02-02	Wild type
EPI_ISL_419951	hCoV-19/Australia/VIC258/2020	2020-03-21	Wild type
EPI_ISL_420037	hCoV-19/Algeria/G0860_2262/2020	2020-03-02	Wild type
EPI_ISL_420059	hCoV-19/France/IDF3165/2020	2020-03-21	Wild type
EPI_ISL_420134	hCoV-19/Norway/1495/2020	2020-02-28	Wild type
EPI_ISL_420152	hCoV-19/Norway/2088/2020	2020-03-17	Wild type
EPI_ISL_420428	hCoV-19/Belgium/VM-0325171/2020	2020-03-25	Wild type
EPI_ISL_420455	hCoV-19/Hong_Kong/HKPU102_2802/2020	2020-03-04	Wild type
EPI_ISL_420636	hCoV-19/England/20136085404/2020	2020-03-24	Wild type
EPI_ISL_420854	hCoV-19/DRC/521/2020	2020-03-25	Wild type
EPI_ISL_421221	hCoV-19/Hangzhou/HZCDC6789/2020	2020-03-15	Wild type
EPI_ISL_421238	hCoV-19/Nanchang/JX174/2020	2020-01-29	Wild type
EPI_ISL_421239	hCoV-19/Shangrao/JX105/2020	2020-01-26	Wild type
EPI_ISL_421246	hCoV-19/Nanchang/JXN3T4/2020	2020-02-26	Wild type
EPI_ISL_421251	hCoV-19/Shangrao/JX1176/2020	2020-02-03	Wild type
EPI_ISL_421260	hCoV-19/Xinyu/JX124/2020	2020-01-26	Wild type
EPI_ISL_421653	hCoV-19/Latvia/01/2020	2020-03-25	Wild type
EPI_ISL_421737	hCoV-19/Luxembourg/LNS2334563/2020	2020-03-17	Wild type
EPI_ISL_421968	hCoV-19/England/20146005304/2020	2020-04-01	Wild type
EPI_ISL_422055	hCoV-19/Wales/PHWC-25E47/2020	2020-03-25	Wild type
EPI_ISL_422427	hCoV-19/Kuwait/KU008/2020	2020-03-16	Wild type
EPI_ISL_422615	hCoV-19/Netherlands/NA_306/2020	2020-04-01	Wild type
EPI_ISL_423250	hCoV-19/England/201360220/2020	2020-03-25	Wild type
EPI_ISL_423251	hCoV-19/England/201360221/2020	2020-03-25	Wild type
EPI_ISL_424580	hCoV-19/Iceland/560/2020	2020-03-27	Wild type
EPI_ISL_424581	hCoV-19/Iceland/561/2020	2020-03-28	Wild type
EPI_ISL_424582	hCoV-19/Iceland/562/2020	2020-03-27	Wild type
EPI_ISL_424583	hCoV-19/Iceland/563/2020	2020-03-28	Wild type
EPI_ISL_424609	hCoV-19/Iceland/589/2020	2020-03-29	Wild type
EPI_ISL_424658	hCoV-19/Belgium/ULG-10049/2020	2020-04-05	Wild type
EPI_ISL_424667	hCoV-19/Mexico/EdoMex-InDRE_03/2020	2020-03-04	Wild type
EPI_ISL_425118	hCoV-19/Korea/KCDC2002/2020	2020-01-30	Wild type
EPI_ISL_425199	hCoV-19/Spain/Valencia75/2020	2020-03-19	Wild type
EPI_ISL_425794	hCoV-19/Scotland/CVR635/2020	2020-03-25	Wild type
EPI_ISL_426166	hCoV-19/Korea/KCDC2005/2020	2020-02-04	Wild type
EPI_ISL_426173	hCoV-19/Korea/KCDC2009/2020	2020-02-07	Wild type
EPI_ISL_426179	hCoV-19/India/c31/2020	2020-03-02	Wild type
EPI_ISL_426414	hCoV-19/India/GBRC1/2020	2020-04-05	Wild type
EPI_ISL_426714	hCoV-19/Australia/VIC414/2020	2020-03-26	Wild type
EPI_ISL_426780	hCoV-19/Australia/VIC510/2020	2020-03-23	Wild type
EPI_ISL_426895	hCoV-19/Czech_Republic/IAB_21/2020	2020-03-27	Wild type
EPI_ISL_426967	hCoV-19/Australia/VIC671/2020	2020-03-27	138-nt deletion
EPI_ISL_427052	hCoV-19/Australia/VIC777/2020	2020-03-30	138-nt deletion
EPI_ISL_427085	hCoV-19/Australia/VIC812/2020	2020-04-02	Wild type
EPI_ISL_427116	hCoV-19/Australia/VIC857/2020	2020-04-05	Wild type
EPI_ISL_427305	hCoV-19/Brazil/SC-766/2020	2020-03-10	Wild type
EPI_ISL_427391	hCoV-19/Turkey/GLAB-CoV008/2020	2020-04-13	Wild type
EPI_ISL_427394	hCoV-19/Taiwan/TSGH-07/2020	2020-03-22	Wild type
EPI_ISL_427408	hCoV-19/Qatar/QA13/2020	2020-03-23	Wild type
EPI_ISL_427427	hCoV-19/USA/WI-UW-120/2020	2020-04-13	Wild type
EPI_ISL_427428	hCoV-19/USA/WI-UW-121/2020	2020-04-11	Wild type
EPI_ISL_427430	hCoV-19/USA/WI-UW-123/2020	2020-04-11	Wild type
EPI_ISL_427439	hCoV-19/USA/WI-UW-132/2020	2020-04-10	Wild type
EPI_ISL_427445	hCoV-19/USA/WI-UW-138/2020	2020-04-12	Wild type
EPI_ISL_427449	hCoV-19/USA/WI-UW-142/2020	2020-04-10	Wild type

EPI_ISL_427452	hCoV-19/USA/WI-UW-145/2020	2020-04-10	Wild type
EPI_ISL_427460	hCoV-19/USA/WI-UW-153/2020	2020-04-09	Wild type
EPI_ISL_427461	hCoV-19/USA/WI-UW-154/2020	2020-04-12	Wild type
EPI_ISL_428234	hCoV-19/Poland/1105973/2020	2020-03-19	Wild type
EPI_ISL_428267	hCoV-19/USA/WI-UW-171/2020	2020-03-19	Wild type
EPI_ISL_428348	hCoV-19/France/GE3372/2020	2020-03-20	Wild type
EPI_ISL_428398	hCoV-19/USA/CT-Yale-095/2020	2020-04-08	Wild type
EPI_ISL_428488	hCoV-19/Taiwan/8/2020	2020-01-31	Wild type
EPI_ISL_428676	hCoV-19/Spain/Madrid_LP19_4952/2020	2020-03-07	Wild type
EPI_ISL_428822	hCoV-19/Singapore/43/2020	2020-02-16	382-nt deletion
EPI_ISL_428830	hCoV-19/Singapore/51/2020	2020-02-27	382-nt deletion
EPI_ISL_428857	hCoV-19/Gambia/GC19-029/2020	2020-04-20	Wild type
EPI_ISL_428885	hCoV-19/Russia/Samara-73406/2020	2020-03-20	Wild type
EPI_ISL_428916	hCoV-19/Russia/Chechenya-83803/2020	2020-03-26	Wild type
EPI_ISL_428928	hCoV-19/Poland/PL_P5/2020	2020-03-30	Wild type
EPI_ISL_428960	hCoV-19/Luxembourg/LNS6326231/2020	2020-04-02	Wild type
EPI_ISL_429098	hCoV-19/Guangzhou/GZMU0054/2020	2020-01-29	Wild type
EPI_ISL_429105	hCoV-19/Guangzhou/GZMU0055/2020	2020-01-29	Wild type
EPI_ISL_429179	hCoV-19/Thailand/Bangkok-0043/2020	2020-03-28	Wild type
EPI_ISL_429210	hCoV-19/Switzerland/GE8147/2020	2020-03-16	Wild type
EPI_ISL_429220	hCoV-19/Switzerland/GE6065/2020	2020-04-06	Wild type
EPI_ISL_429681	hCoV-19/Brazil/CV21/2020	2020-03-16	Wild type
EPI_ISL_429722	hCoV-19/Luxembourg/LNS6137379/2020	2020-04-01	Wild type
EPI_ISL_429852	hCoV-19/Lishui/LS111/2020	2020-01-25	Wild type
EPI_ISL_429854	hCoV-19/Lishui/LS557/2020	2020-02-01	Wild type
EPI_ISL_429861	hCoV-19/Turkey/HSGM-8968/2020	2020-03-22	Wild type
EPI_ISL_429968	hCoV-19/France/HF1463/2020	2020-02-21	Wild type
EPI_ISL_429997	hCoV-19/Jordan/SR-037/2020	2020-03-16	Wild type
EPI_ISL_430440	hCoV-19/Malaysia/IMR_WC1170/2020	2020-03-05	Wild type
EPI_ISL_430441	hCoV-19/Malaysia/IMR_WC1097/2020	2020-02-29	Wild type
EPI_ISL_430443	hCoV-19/Malaysia/IMR_WC085/2020	2020-01-28	Wild type
EPI_ISL_430576	hCoV-19/Australia/VIC1114/2020	2020-04-07	Wild type
EPI_ISL_430741	hCoV-19/Beijing/BJ782/2020	2020-02-10	Wild type
EPI_ISL_430742	hCoV-19/Beijing/BJ251/2020	2020-01-29	Wild type
EPI_ISL_430799	hCoV-19/Argentina/PAIS_A007/2020	2020-03-30	Wild type
EPI_ISL_430801	hCoV-19/Argentina/PAIS_A009/2020	2020-03-31	Wild type
EPI_ISL_430803	hCoV-19/Argentina/PAIS_A012/2020	2020-04-01	Wild type
EPI_ISL_430804	hCoV-19/Argentina/PAIS_A013/2020	2020-04-01	Wild type
EPI_ISL_430816	hCoV-19/Argentina/PAIS_A025/2020	2020-04-18	Wild type
EPI_ISL_430817	hCoV-19/Argentina/PAIS_A026/2020	2020-04-18	Wild type
EPI_ISL_431782	hCoV-19/Fujian/IM3520014T/2020	2020-03-22	Wild type
EPI_ISL_431975	hCoV-19/Wales/PHWC-275AA/2020	2020-03-31	Wild type
EPI_ISL_432624	hCoV-19/England/SHEF-D10F9/2020	2020-03-24	Wild type
EPI_ISL_433446	hCoV-19/Scotland/EDB1433/2020	2020-04-12	Wild type
EPI_ISL_433631	hCoV-19/Scotland/CVR1613/2020	2020-04-05	Wild type
EPI_ISL_434535	hCoV-19/Costa_Rica/02/2020	2020-03-16	Wild type
EPI_ISL_434545	hCoV-19/Puerto_Rico/CDC-S5/2020	2020-03-23	Wild type
EPI_ISL_434572	hCoV-19/Czech_Republic/2308/2020	2020-04-14	Wild type
EPI_ISL_434669	hCoV-19/Sweden/20-07390/2020	2020-04-15	Wild type
EPI_ISL_435032	hCoV-19/DRC/1516/2020	2020-04-09	Wild type
EPI_ISL_435047	hCoV-19/Kazakhstan/NCB-3/2020	2020-03-25	Wild type
EPI_ISL_435123	hCoV-19/United_Arab_Emirates/L0484/2020	2020-03-12	Wild type
EPI_ISL_435134	hCoV-19/United_Arab_Emirates/L4280/2020	2020-02-08	Wild type
EPI_ISL_435137	hCoV-19/United_Arab_Emirates/L5630/2020	2020-01-29	Wild type
EPI_ISL_435316	hCoV-19/Vietnam/OUCRU0985/2020	2020-03-17	Wild type
EPI_ISL_435403	hCoV-19/Hungary/SRC-00066/2020	2020-03-20	Wild type
EPI_ISL_435694	hCoV-19/Singapore/89/2020	2020-04-22	Wild type
EPI_ISL_436226	hCoV-19/Spain/Valencia131/2020	2020-03-17	Wild type

EPI_ISL_436330	hCoV-19/Spain/Valencia235/2020	2020-03-26	Wild type
EPI_ISL_436732	hCoV-19/Italy/TE26425/2020	2020-04-27	Wild type
EPI_ISL_437187	hCoV-19/Indonesia/EJ-ITD853Sp/2020	2020-03-25	Wild type
EPI_ISL_437192	hCoV-19/Indonesia/JKT-EIJK04/2020	2020-04-01	Wild type
EPI_ISL_437221	hCoV-19/Germany/BAV-MVP0021/2020	2020-04-06	Wild type
EPI_ISL_437237	hCoV-19/Germany/BAV-MVP0038/2020	2020-03-23	Wild type
EPI_ISL_437300	hCoV-19/Austria/Graz-MUG11/2020	2020-04-14	Wild type
EPI_ISL_437322	hCoV-19/Turkey/HSGM-1009/2020	2020-03-19	Wild type
EPI_ISL_437626	hCoV-19/India/GMC-KP1125/2020	2020-03-24	Wild type
EPI_ISL_437643	hCoV-19/Denmark/ALAB-SSI-1334/2020	2020-04-02	Wild type
EPI_ISL_437708	hCoV-19/Saudi_Arabia/KAUST-Makkah193/2020	2020-04-14	Wild type
EPI_ISL_437714	hCoV-19/Saudi_Arabia/KAUST-Makkah204/2020	2020-04-14	Wild type
EPI_ISL_437717	hCoV-19/Saudi_Arabia/KAUST-Madinah213/2020	2020-04-14	Wild type
EPI_ISL_437720	hCoV-19/Saudi_Arabia/KAUST-Makkah218/2020	2020-04-16	Wild type
EPI_ISL_437887	hCoV-19/Greece/220_35357/2020	2020-03-18	Wild type
EPI_ISL_437941	hCoV-19/Austria/CeMM0071/2020	2020-03-11	Wild type
EPI_ISL_438766	hCoV-19/Scotland/CVR1113/2020	2020-03-29	Wild type
EPI_ISL_438971	hCoV-19/Japan/Donner27/2020	2020-04-24	Wild type
EPI_ISL_441401	hCoV-19/Northern_Ireland/NIRE-102304/2020	2020-03-23	Wild type
EPI_ISL_441698	hCoV-19/Northern_Ireland/NIRE-FA968/2020	2020-04-06	Wild type
EPI_ISL_442523	hCoV-19/Iran/KHGRC-1.1-IPI-8206/2020	2020-03-09	Wild type
EPI_ISL_443168	hCoV-19/Saudi_Arabia/KAUST-Madinah24/2020	2020-03-29	Wild type
EPI_ISL_443286	hCoV-19/France/IDF5657/2020	2020-04-02	Wild type
EPI_ISL_444025	hCoV-19/USA/CA-CZB-1091/2020	2020-05-01	Wild type
EPI_ISL_444471	hCoV-19/India/GBRC40/2020	2020-04-29	Wild type
EPI_ISL_444495	hCoV-19/Canada/QC_AA3/2020	2020-04-05	Wild type
EPI_ISL_444905	hCoV-19/Denmark/ALAB-SSI-787/2020	2020-03-30	Wild type
EPI_ISL_444972	hCoV-19/Spain/Barcelona_VH6819/2020	2020-04-05	Wild type
EPI_ISL_445000	hCoV-19/Guam/GU_NHG_03/2020	2020-03-20	Wild type
EPI_ISL_445075	hCoV-19/Luxembourg/LNS1586475/2020	2020-05-08	Wild type
EPI_ISL_445244	hCoV-19/Bangladesh/Akbiomed_01/2020	2020-04-25	Wild type
EPI_ISL_445309	hCoV-19/Chile/Santiago_30/2020	2020-03-16	Wild type
EPI_ISL_445351	hCoV-19/Chile/Santiago_55/2020	2020-04-02	Wild type
EPI_ISL_445954	hCoV-19/Wales/PHWC-2CB67/2020	2020-04-01	Wild type
EPI_ISL_447018	hCoV-19/Thailand/Bangkok-0077/2020	2020-04-01	Wild type
EPI_ISL_447033	hCoV-19/India/GBRC58/2020	2020-05-03	Wild type
EPI_ISL_447043	hCoV-19/India/GBRC67a/2020	2020-05-03	Wild type
EPI_ISL_447054	hCoV-19/Romania/279068/2020	2020-04-30	Wild type
EPI_ISL_447056	hCoV-19/Georgia/Tb-6572/2020	2020-04-29	Wild type
EPI_ISL_447123	hCoV-19/Belgium/ULG-9535/2020	2020-03-21	Wild type
EPI_ISL_447153	hCoV-19/Belgium/ULG-10158/2020	2020-04-18	Wild type
EPI_ISL_447262	hCoV-19/Israel/990333068/2020	2020-03-24	Wild type
EPI_ISL_447334	hCoV-19/Israel/2115990/2020	2020-04-16	Wild type
EPI_ISL_447524	hCoV-19/Spain/Valencia605/2020	2020-04-01	Wild type
EPI_ISL_447536	hCoV-19/India/GBRC78a/2020	2020-05-05	Wild type
EPI_ISL_447551	hCoV-19/India/GBRC89/2020	2020-04-25	Wild type
EPI_ISL_447621	hCoV-19/Taiwan/NTU27/2020	2020-04-13	Wild type
EPI_ISL_447734	hCoV-19/Colombia/GUR-0072/2020	2020-03-26	Wild type
EPI_ISL_447759	hCoV-19/Colombia/GUV-92061/2020	2020-03-31	Wild type
EPI_ISL_447786	hCoV-19/Colombia/GVI-93534/2020	2020-04-03	Wild type
EPI_ISL_448001	hCoV-19/England/CAMB-1AD72D/2020	2020-05-01	Wild type
EPI_ISL_448357	hCoV-19/England/NORW-E9C54/2020	2020-04-30	Wild type
EPI_ISL_448928	hCoV-19/Northern_Ireland/NIRE-FAE4B/2020	2020-03-26	Wild type
EPI_ISL_449321	hCoV-19/Scotland/EDB4682/2020	2020-05-09	Wild type
EPI_ISL_450188	hCoV-19/Jordan/SR-0336/2020	2020-04-06	Wild type
EPI_ISL_450209	hCoV-19/Germany/BavPat2-ChVir984-ChVir1017/2020	2020-01-30	Wild type
EPI_ISL_450254	hCoV-19/Russia/StPetersburg-RII7501S/2020	2020-04-20	Wild type
EPI_ISL_450294	hCoV-19/Poland/IHG_PAS_1_69/2020	2020-04-11	Wild type

EPI_ISL_450299	hCoV-19/South_Africa/R07552/2020	2020-03-31	Wild type
EPI_ISL_450301	hCoV-19/South_Africa/R07719/2020	2020-04-01	Wild type
EPI_ISL_450339	hCoV-19/Bangladesh/BARJ_CVASU_CTG_501/2020	2020-05-10	Wild type
EPI_ISL_450343	hCoV-19/Bangladesh/BARJ_CVASU_CTG_511/2020	2020-05-09	345-nt deletion
EPI_ISL_450344	hCoV-19/Bangladesh/BARJ_CVASU_CTG_517/2020	2020-05-03	345-nt deletion
EPI_ISL_450509	hCoV-19/Lebanon/S2_759/2020	2020-03-15	Wild type
EPI_ISL_450522	hCoV-19/Latvia/022/2020	2020-04-28	Wild type
EPI_ISL_450526	hCoV-19/Poland/1109500/2020	2020-03-29	Wild type
EPI_ISL_450799	hCoV-19/JAM/JM-CDC-7286/2020	2020-03-17	Wild type
EPI_ISL_450808	hCoV-19/Sweden/20-06665/2020	2020-03-31	Wild type
EPI_ISL_450832	hCoV-19/Sweden/20-51167/2020	2020-05-04	Wild type
EPI_ISL_451076	hCoV-19/Sichuan/SC-WCH2-006/2020	2020-02-08	Wild type
EPI_ISL_451155	hCoV-19/India/GBRC110/2020	2020-05-03	Wild type
EPI_ISL_451162	hCoV-19/India/GBRC117/2020	2020-05-03	Wild type
EPI_ISL_452497	hCoV-19/Spain/COV001404/2020	2020-03-19	62-nt deletion
EPI_ISL_452530	hCoV-19/Spain/COV001371/2020	2020-03-13	62-nt deletion
